# Progression of Left Ventricular Aneurysm to Pseudoaneurysm on Serial Imaging

**DOI:** 10.1016/j.cjco.2024.04.002

**Published:** 2024-04-10

**Authors:** Yoshito Kadoya, Alexander Dick, Hassan Mir, Luc Beauchesne, D. Ian Paterson

**Affiliations:** a Division of Cardiology, University of Ottawa Heart Institute, University of Ottawa, Ottawa, Ontario, Canada

**Left ventricular (LV) aneurysms and pseudoaneurysm are 2 serious complications arising from acute myocardial infarction (MI) and are associated with an increased risk of morbidity and mortality.** **^1^** **Although modern multimodal imaging techniques have improved the identification of incidental LV pseudoaneurysms in asymptomatic patients during the latent phase of MI, the pathogenesis of pseudoaneurysms remains poorly understood. We describe the case of a 75-year-old man with late-presenting MI, LV aneurysm, and massive thrombus. Follow-up cardiac imaging at 6 weeks identified a new pseudoaneurysm arising from the aneurysm and near resolution of the thrombus.**

## Case

A 75-year-old male patient with hypertension, dyslipidemia, diabetes, and active smoking presented to the emergency department with a 3-month history of nonproductive cough and bilateral lower-extremity edema. The patient had no prior history of cardiac disease and denied chest pain or dyspnea. On presentation, he was afebrile, with a blood pressure of 128/88 mm Hg, a heart rate of 99 beats per minute, and an oxygen saturation of 99% on room air. Physical examination was notable for an elevated jugular venous pressure and 2+ pitting edema of both lower legs. Laboratory results revealed a high-sensitivity troponin T level of 284 ng/L (reference range: < 14 ng/L), an N-terminal pro-B-type natriuretic peptide level of 34,422 ng/L (reference range: < 300 ng/L), and a positive nasopharyngeal polymerase chain reaction test for severe acute respiratory syndrome (SARS)-CoV-2. A chest radiograph showed opacification in the left lower lobe, with an associated small left pleural effusion consistent with pneumonia. An electrocardiogram demonstrated Q waves in leads V1-4, with ST-segment elevation in leads V3-6. The patient was admitted with a diagnosis of community-acquired pneumonia secondary to COVID-19 infection and congestive heart failure potentially due to a silent MI.

**Figure 1:**
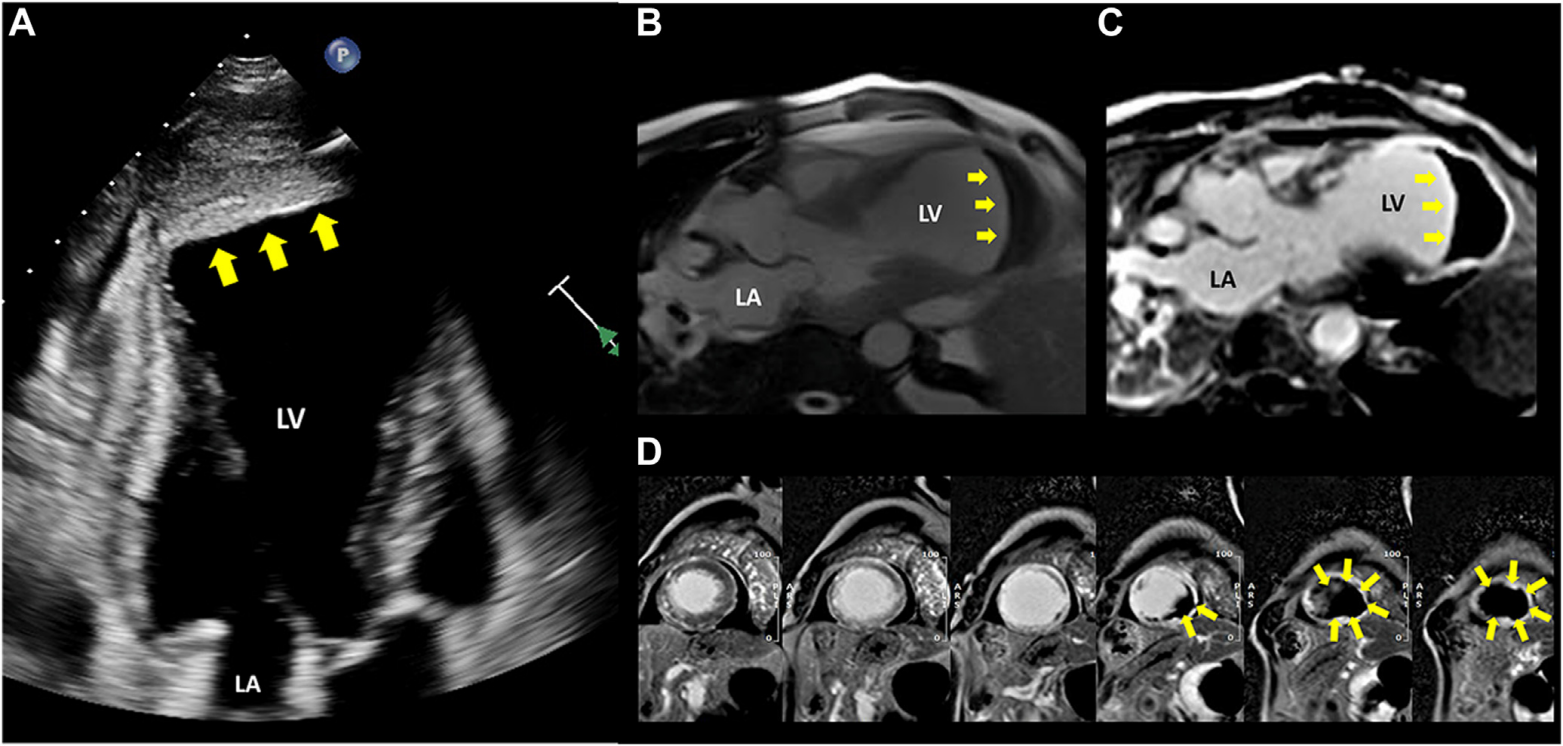
Transthoracic echocardiogram and cardiac magnetic resonance imaging at presentation. (**A**) Apical 3-chamber view on the transthoracic echocardiogram demonstrates a left ventricular (LV) apical aneurysm with substantial LV thrombus (**yellow arrows**). (**B**) Three-chamber steady-state free precession cine imaging showing a true LV apical aneurysm with a giant LV thrombus (**yellow arrows**) with indexed LV end-diastolic and end-systolic volumes of 201 mL/m^2^ and 179 mL/m^2^, respectively. (**C,D**) Late gadolinium enhancement imaging demonstrating the thrombus (**yellow arrows**) and transmural myocardial enhancement of the LV apex—(**C**) 3-chamber view; (**D**) short-axis views from mid-ventricular to apex. LA, left atrium; (in figure) LV, left ventricle.

He was started on dual-antiplatelet therapy, furosemide 40 mg intravenous daily, guideline-directed heart failure therapy, and antibiotics (ceftriaxone 2 g every 24 hours, and doxycycline 100 mg every 12 hours). On day 6 of admission, a transthoracic echocardiogram found severe LV dysfunction, with a LV ejection fraction of 20% and a large anteroapical aneurysm and mural thrombus (Fig. 1A; Video 1 , view video online). Subsequent cardiac magnetic resonance imaging (CMR) confirmed a broad-based LV aneurysm filled with a large volume of thrombus (Fig. 1, B-D; Videos 2 and 3 , view videos online). Coronary angiography revealed 90% stenosis in the proximal and mid left anterior descending artery, and 80% stenosis in the first diagonal branch. Given the nonviable myocardium identified on CMR, revascularization was not performed. Anticoagulation with intravenous heparin was initiated and the patient was transitioned to apixaban 5 mg twice daily 4 days later. His heart failure and pneumonia improved with medical therapy; however, other complications, including acute kidney injury and delirium, prolonged his hospital stay.

**Figure 2:**
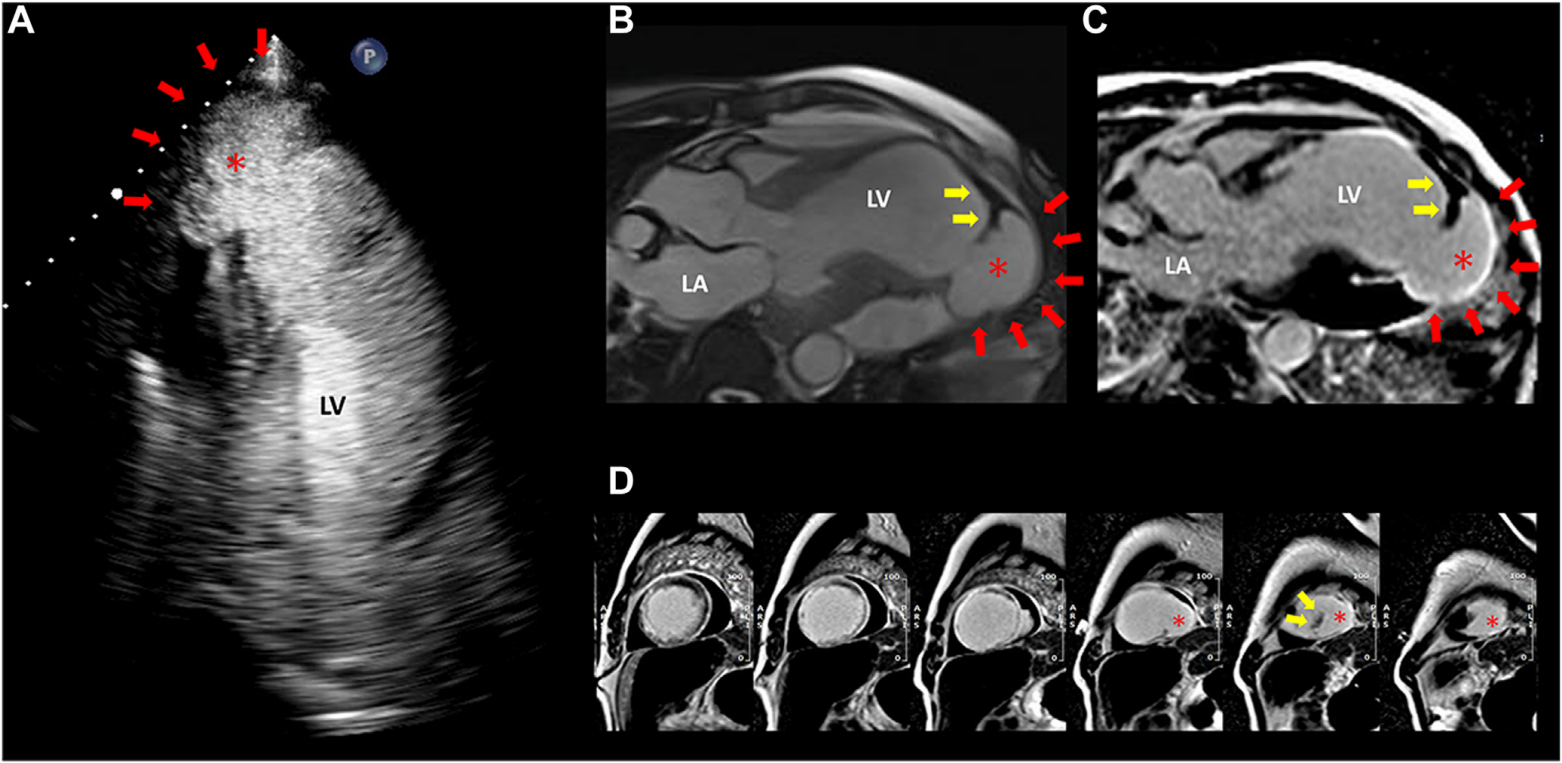
Follow-up transthoracic echocardiogram and cardiac magnetic resonance imaging. (**A**) Apical 3-chamber view on the transthoracic echocardiogram with an ultrasound-enhancing contrast showing a morphologic change of the left ventricular (LV) aneurysm suspicious for pseudoaneurysm (**asterisk and red arrows**). The imaging hallmarks of pseudoaneurysm include a saccular-shaped structure with a narrow neck and an abrupt transition in wall thickness and angulation at its origin. (**B**) Three-chamber steady state free precession cine imaging demonstrating pseudoaneurysm formation (**asterisk and red arrows**) in the apical inferolateral segment, and remnant LV thrombus (**yellow arrows**) with indexed LV end-diastolic and end-systolic volumes of 232 mL/m^2^ and 184 mL/m^2^, respectively. (**C,D**) Late gadolinium enhancement imaging demonstrating the pseudoaneurysm (**asterisk and red arrows**) and remnant thrombus (**yellow arrows**); (**C**) 3-chamber view; (**D**) short-axis views from mid-ventricular to apex). LA, left atrium; (in figure) LV, left ventricle.

A follow-up transthoracic echocardiogram at 6 weeks showed partial resolution of the LV thrombus, but an increase in the size and shape of the LV aneurysm was concerning for presence of a pseudoaneurysm (Fig. 2A; Video 4 , view video online). Further evaluation with CMR revealed disruption of the LV apical aneurysm and new inferolateral outpouching and extreme wall thinning, consistent with a pseudoaneurysm and contained LV rupture (Fig. 2, B-D; Videos 5 and 6 , view videos online). The patient was deemed to be at high risk for surgical repair, given his severe LV dysfunction and noncardiac comorbidities. Anticoagulation was discontinued, and the patient was transferred to a long-term care hospital on hospital day 75. No follow-up imaging is available; however, he remains stable at 6 months after his initial presentation.

## Discussion

LV pseudoaneurysm is a rare but serious complication of acute MI that is characterized by rupture of the myocardial wall, with containment by the pericardium, thrombus, or adhesions.^1^ Unlike true LV aneurysms, which occur from a weakened myocardial wall that retains all myocardial layers, pseudoaneurysms lack both the endocardium and myocardium.^1^ Pseudoaneurysms are associated with a significantly increased risk of cardiac rupture, previously estimated at 30%-46%.^1^ Although modern multimodal imaging techniques have improved the identification of incidental LV pseudoaneurysms in asymptomatic patients during the latent phase of MI, the pathogenesis of pseudoaneurysms remains poorly understood.

To date, a pathogenetic relationship between LV thrombus and subsequent cardiac rupture or pseudoaneurysm formation has not been established. However, emerging evidence from preclinical and clinical studies suggests a possible association. Ma et al. have hypothesized that MI-related LV thrombus arises from endocardial rupture and exposure of infarcted tissue, triggering platelet activation and aggregation at the site of injury.^2^ This intramural, platelet-rich thrombus may propagate into the LV cavity and form an intraluminal thrombus. Furthermore, the LV thrombus itself may initiate a pathogenic milieu conducive to cardiac rupture and pseudoaneurysm formation. In patients with abdominal aortic aneurysms, intraluminal thrombus has been associated with aneurysm growth and early rupture.^3^ One possible mechanism is that, although the thrombus initially reduces wall stress, proteolytic enzymes eventually cause destabilization of wall integrity and contribute to the risk of aortic aneurysm rupture.^4^ However, the potential pathogenic role of thrombus potentiating LV pseudoaneurysm remains speculative. We also cannot exclude the possibility that an early-stage pseudoaneurysm existed at presentation, which highlights the potential need for early follow-up imaging in patients with late-presenting MI.

In our patient with late-presenting MI and large LV thrombus, we observed a silent transformation of LV aneurysm to pseudoaneurysm. We believe that this case is the first demonstrating this progression on serial multimodal imaging. Future studies should be performed to better characterize post-MI LV thrombus burden as a potential mechanism of pseudoaneurysm formation.

**Novel Teaching Points**

• LV pseudoaneurysm is a serious complication of acute MI, with a significantly increased risk of cardiac rupture.
• The pathogenesis of LV pseudoaneurysm remains poorly understood; however, LV thrombus may serve as a potential trigger for its formation.
• Careful follow-up with multimodal imaging is useful to detect subclinical transformation to LV pseudoaneurysm.

## Supplementary Material

Video 1 Transthoracic echocardiogram apical three-chamber view at presentation.

Video 2 Cardiac magnetic resonance imaging three-chamber cine (balanced steady-state free precession cine sequence) at presentation.

Video 3 Cardiac magnetic resonance short-axis cine at presentation.

Video 4 Transthoracic echocardiogram apical three-chamber view with an ultrasound-enhancing agent at 6 weeks.

Video 5 Cardiac magnetic resonance three-chamber cine at 6 weeks.

Video 6 Cardiac magnetic resonance short-axis cine at 6 weeks.
